# Supplementary material for: Learning curve and surgical outcome of robotic assisted colorectal surgery with ERAS program
Source: Sci Rep. 2022 Nov 29;12:20566. doi: 10.1038/s41598-022-24665-w (PMC9709162; doi:10.1038/s41598-022-24665-w)
Supplement: Supplementary file 1 — Supplementary Information 1. [file 41598_2022_24665_MOESM1_ESM.zip › LSC Raw data-Ñ[▒K/140/N_ñw╝╨░O▒Kñσ.pdf]

W83 / W83

臺中榮民總醫院
手術室護理記錄

[redacted]

基本資料

- 室別： 二醫手術室-31室，手術科別： CRS，手術類別： 常規，日期： 2021/10/06
- 進入時間： 1015 ； 麻醉開始時間： 1015 ； Time Out時間： 1125 ； 劃刀時間： 1128 ； 手術結束時間： 1555 送出時間： 1620 ；
- 麻醉方式： GE
  診斷： Malignant tumor of T-colon
  手術名稱： Laparoscopic right colectomy and anastomosis
  手術局部用藥： Nil
  術前預防性抗生素：
  - 抗生素名稱一： Veterin 1GM IVD/由麻醉護理給予
  - 時間： 11115
  - 上次簽章: 林采儀

  ，追加預防性抗生素：
  - 抗生素名稱一： 1GM IVD/由麻醉護理給予
  - 時間： 1530
  - 上次簽章: 林采儀
- 手術醫師： 林俊余，林志安，林順鴻
- 麻醉醫師： VS 張思玲
-
  - 刷手護士A ： 彭馨慧，時間： 1015，刷手護士B ： 吳明柔，時間： 1015，接替刷手一： 許育庭，時間： 1200，接替刷手二： 彭馨慧，時間： 1500
  - 流動護士A ： 林采儀，時間： 1015，接替流動一： 吳明柔，時間： 1100，接替流動二： 林采儀，時間： 1130
- 更多接替及流動護士： 接替流動三： 黃仁慧，時間： 1600

皮膚狀況

A-V Shunt： 無
術前： 完整
記錄者： 上次簽章: 吳明柔
術後： 完整
記錄者： 上次簽章: 林采儀

手術準備

手術臥式 單一手術臥式

-
  - 手術臥式： 仰臥
  - 使用附件： 溫毯 38 ℃，頭圈
  - 皮膚準備： 10%Aq Betadine，Hibiscrub，2% Chlorhexidine
  - 電燒： 使用電燒: 電極片位置： 其它： 床上迴路電極墊，記錄者： 上次簽章: 吳明柔

止血帶T： 無

植入物

- 植入物名稱/號碼/數量/部位： Ethicon ligaclip/LOT V94A8Y/20釘/colon
  Ethicon endo-surgery 60/LOT V9455M/參釘/colon

，記錄者： 上次簽章: 林采儀

標本送檢

- 細菌培養 無
- 細胞學 無
- 冰凍切片 無
- 病理標本

組織名稱一： R't side colon

- 截肢 無

標本交付 標本： 壹 袋 , 標本： 零 盒 , 標本： 零 瓶 , 細胞學： 零 管 , Culture Tube： 零 管 , 被交付醫師： R 林順鴻 ,

- 記錄者： 上次簽章: 林采儀
- 時間： 1510

傷口術後敷料

傷口敷料： 傷口敷料： 無

- 請於圖上標示部位：

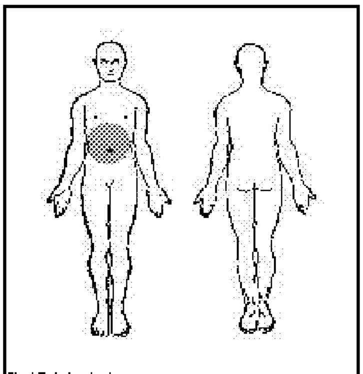

- 傷口敷料： Mepore覆蓋

導管

導管	天數	導管資訊	插入日期	插入時間	更換日期	拔除日期	拔除時間	拔除原因	預計拔除/更換	預計拔除/更換日期
Foley(1)	2	號碼:16 Fr.; 部位:膀胱;	2021/10/06	1050						
Penrose Drain(1)	2	號碼:12 mm.; 部位:肝下;	2021/10/06	1445						
CVC(1)	2	號碼:8 ; 深度:15cm; 部位:jugular (右); 象限:RU;	2021/10/06	1105						
Sump NG Tube(1)	2	號碼:16 Fr.; 深度:60cm; 部位:胃;	2021/10/06	1100						
A-line(1)	2	號碼:20 號; 部位:左手;	2021/10/06	1050						
Permanent Pacemaker (1)	291	部位:左胸; 註記:DDD MODE ; RATE 60-120次/分;	2015/03/10	0900	2020/12/21 0900					

用物計數

- 手術中出血量： Minimal c.c.
- 手術中尿量： 300 c.c.
-
    - 其它： 無

用物計數： 單一切口

- 1.器械： 正確 , 2.縫針： 正確 , 3.刀片 ： 正確 , 4.紗布： 正確 , 5.腹部墊： 未使用 , 6.紗球： 未使用 , 7.PEANUTS ： 未使用 , 8.紗條： 未使用 , 9.絲帶： 未使用 , 10.針頭： 未使用 , 11.Vessel loop： 未使用 , 12.Boots： 未使用 , 13.Jelco： 未使用 , 14.棉條： 未使用 ,
    - 第一次：
        - 刷手護士 ： 許育庭
        - 流動護士： 上次簽章: 林采儀
        - 時間： 1440

,

-
    - 第二次：
        - 刷手護士 ： 許育庭
        - 流動護士： 上次簽章: 林采儀
        - 時間： 1500

,

- 第三次：
  - 刷手護士 ： 彭馨慧
  - 流動護士： 上次簽章: 林采儀
  - 時間： 1520

備註

備註： NIL

- 記錄者： 上次簽章: 黃仁慧
- 時間： 1620

填寫護理人員 林采儀

正式摘要

W83 / W83

doctorRank:B
